# Morphological and Molecular Responses of *Lateolabrax maculatus* Skeletal Muscle Cells to Different Temperatures

**DOI:** 10.3390/ijms23179812

**Published:** 2022-08-29

**Authors:** Jingru Zhang, Haishen Wen, Xin Qi, Yonghang Zhang, Ximeng Dong, Kaiqiang Zhang, Meizhao Zhang, Jifang Li, Yun Li

**Affiliations:** Key Laboratory of Mariculture, Ministry of Education (KLMME), Ocean University of China, Qingdao 266003, China

**Keywords:** *Lateolabrax maculatus*, skeletal muscle cells, myogenesis, temperatures, primary cell culture, RNA-seq

## Abstract

Temperature strongly modulates muscle development and growth in ectothermic teleosts; however, the underlying mechanisms remain largely unknown. In this study, primary cultures of skeletal muscle cells of *Lateolabrax maculatus* were conducted and reared at different temperatures (21, 25, and 28 °C) in both the proliferation and differentiation stages. CCK-8, EdU, wound scratch and nuclear fusion index assays revealed that the proliferation, myogenic differentiation, and migration processes of skeletal muscle cells were significantly accelerated as the temperature raises. Based on the GO, GSEA, and WGCNA, higher temperature (28 °C) induced genes involved in HSF1 activation, DNA replication, and ECM organization processes at the proliferation stage, as well as HSF1 activation, calcium activity regulation, myogenic differentiation, and myoblast fusion, and sarcomere assembly processes at the differentiation stage. In contrast, lower temperature (21 °C) increased the expression levels of genes associated with DNA damage, DNA repair and apoptosis processes at the proliferation stage, and cytokine signaling and neutrophil degranulation processes at the differentiation stage. Additionally, we screened several hub genes regulating myogenesis processes. Our results could facilitate the understanding of the regulatory mechanism of temperature on fish skeletal muscle growth and further contribute to utilizing rational management strategies and promoting organism growth and development.

## 1. Introduction

Fish skeletal muscle is an important source of high-quality protein for human food. The growth performance of skeletal muscle determines food production and economic benefit in the aquaculture industry [1]. In contrast to mammals, for which hypertrophy of existing muscle fibers is the primary mechanism of skeletal muscle growth during the postnatal stages, fish skeletal muscle mass grows by increasing both the number and size of muscle fibers, exhibiting an indeterminate growth pattern that combines hyperplasia and hypertrophy throughout its lifespan [2,3]. The generation of muscle and myogenesis involves a series of steps, including the activation, proliferation, and differentiation of muscle stem cells (MSCs, also known as satellite cells), the fusion of precursors to form syncytial myotubes, and the maturation of myotubes to generate contractile myofibers [4,5,6]. The process of muscle formation is highly regulated by many myogenic regulatory factors (MRFs) and signaling molecules [6,7,8,9].

The indeterminate growth observed in most fishes is influenced by environmental factors such as temperature [10], nutrition [11], salinity [12] and oxygen concentrations [13]. Among them, temperature strongly modulates muscle development in ectothermic teleosts [10]. For example, in gilthead sea bream, myogenesis progression and the expression levels of myofibril organization-related genes such as UNC45 and Hsp90α were modified by temperature during the early stages of the life cycle [14]. By using digital morphometry and immunolabeling to quantitatively examine Pax7, myogenin, and H3P, the proliferation and differentiation of muscle precursor cells (MPCs) were proven to be changed by different temperatures, which was an underlying reason for the lasting effects on muscle cellularity and body growth of European pearlfish (*Rutilus frisii meidingeri*) [10]. Recent studies in European sea bass (*Dicentrarchus labrax*) and Senegalese sole (*Solea senegalensis*) have indicated that temperature-dependent muscle growth is regulated by epigenetic factors and that higher temperature increases myogenin expression and improves muscle growth mediated by DNA methylation or miRNA [15,16]. However, the underlying mechanisms of temperature-regulated muscle growth in teleosts remain largely unknown.

The in vitro culture of muscle cells is an effective system for studying the process of myogenesis and investigating the effects of various regulatory factors on muscle formation and growth [17,18]. The murine myoblast cell line C2C12 has been used extensively as an examination model for mammals and has led to numerous important discoveries in the mechanisms of myogenesis [19,20]. As muscle cell lines are unavailable in most species, primary cultures of skeletal muscle cells have become a viable alternative for muscle biology research [21,22], which can facilitate direct investigation of the impact of precisely controlled conditions, such as changes in cultivation temperatures. For instance, studies on cultured myogenic stem cells (satellite cells) in pigs and turkeys demonstrated that temperature significantly changed gene expression patterns in proliferating and differentiating muscle cells and thus had a profound effect on skeletal muscle growth [23,24]. For teleosts, muscle cells have been isolated and cultured in vitro from zebrafish (*Danio rerio*) [25], rainbow trout (*Oncorhynchus mykiss*) [26], and black rockfish (*Sebastes schlegelii*) [22]. Nevertheless, as mentioned above, myogenesis in fish has several unique characteristics compared to that in endothermal mammals and birds, and the regulation of fish skeletal muscle growth and development, especially the underlying mechanisms controlling the response to different temperatures, is still poorly understood and needs to be further investigated.

The spotted sea bass (*Lateolabrax maculatus*) is one of the most important marine-culture fish species in China, with annual production exceeding 190,000 tons in recent years (China Fishery Statistical Yearbook, 2021). Due to its high nutritional value and pleasant taste, spotted sea bass have become increasingly popular and require an improvement in growth as well as muscle mass to meet market demand. Therefore, understanding the principle of myogenesis in spotted sea bass is fundamentally important to explore valuable possibilities for application to fish culture production. Additionally, the rearing range of water temperature of *L. maculatus* is 16–33 °C, and the phenomenon of temperature-dependent growth has been reported in this species [27,28,29], but knowledge of the regulatory mechanism of skeletal muscle growth and development is still lacking. In this study, we employed primary cultures of *L. maculatus* skeletal muscle cells, in combination with comparative transcriptome analysis, to investigate the effects of different temperatures on in vitro skeletal muscle development. The aims of our study were (1) to establish a method for primary cultures of *L. maculatus* skeletal muscle cells in vitro; (2) to evaluate the effects of temperature on the proliferation, differentiation, and migration of cultured *L. maculatus* skeletal muscle cells; (3) to identify the key genes and pathways responsible for the proliferation and differentiation of skeletal muscle cells; and (4) to understand the potential molecular mechanism accounting for the phenotypic differences in skeletal muscle cells at the proliferation and differentiation stages in response to different temperatures. The results from this study will provide the basis for revealing how temperature affects myogenesis in *L. maculatus* and potentially be used to develop effective management strategies to be beneficial to skeletal muscle growth.

## 2. Results

### 2.1. The Effects of Temperature on the Proliferation, Differentiation and Migration of Skeletal Muscle Cells

**Figure 1:**
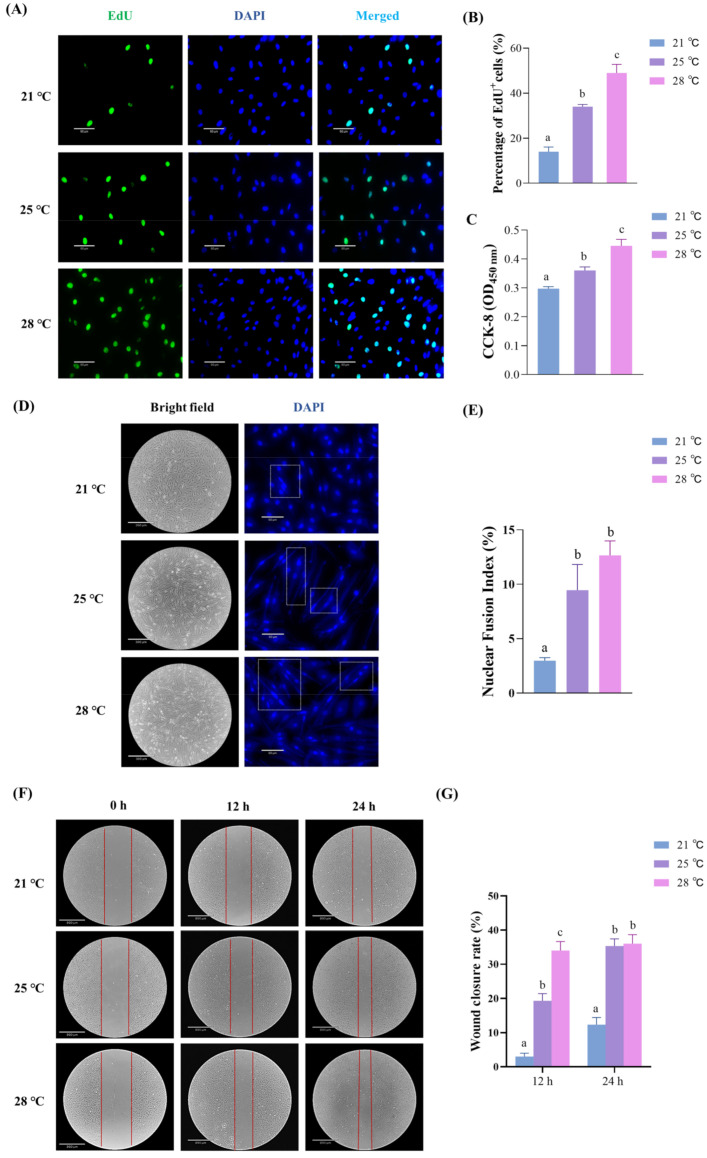
Temperature alters the proliferation, myogenic differentiation and migration of skeletal muscle cells. (**A**) Images of EdU+ cells at 21 °C, 25 °C, and 28 °C. Scale bars = 60 µm. (**B**) The percentage of EdU+ cells was calculated as the ratio of the number of EdU-positive cells to the number of total nuclei at 21 °C, 25 °C, and 28 °C. (**C**) CCK-8 assay of absorbance values at 450 nm wavelength was detected at 21 °C, 25 °C, and 28 °C. (**D**) Nuclei were stained with DAPI at 48 h after inducing differentiation, and white boxes illustrate the multinucleated myotubes with approximately 2–3 nuclei per cell. (**E**) The nuclear fusion index of the myotubes, which was calculated as a ratio of the number of nuclei inside myotubes to the number of total nuclei ×100 after myotube formation at 21 °C, 25 °C, and 28 °C. Scale bars = 60 µm. (**F**) Images of the wound healing assay at 0 h, 12 h, and 24 h reared at 21 °C, 25 °C, and 28 °C, respectively. Scale bars = 890 μm. (**G**) Quantification of the wound closure of the cell migration by ImageJ software (v2.1.0, Bethesda, MD, USA). Data are representative of three independent experiments performed in triplicate. Different letters (a–c) represent significant differences between groups (*p* < 0.05).

In vitro EdU and CCK-8 assays were employed to evaluate the effect of temperature on cell proliferation. The EdU assay showed that the ratio of EdU+ cells was positively correlated with temperature; in comparison with that at 21 °C, the percentage of EdU+ cells cultured at 25 °C and 28 °C increased 2.43-fold and 3.5-fold, respectively (Figure 1A,B). For the CCK-8 assay, the absorbance was determined at a wavelength of 450 nm to assess cell proliferation. As shown in Figure 1C, the OD values at 450 nm increased with increasing temperature (28 °C > 25 °C > 21 °C). These results indicated that the proliferative rate of skeletal muscle cells was increased as the temperature rises.

The effect of temperature on differentiation was revealed by the NFI assay. As shown in Figure 1D,E, although no significant difference was observed between cells cultured at 25 °C and 28 °C, the nuclear fusion index (NFI) values at 25 °C and 28 °C were remarkably higher than those at 21 °C. Notably, a number of multinucleated myotubes (with 3 nuclei per cell) were observed only in cells cultured at 28 °C (Figure 1D). These results indicated that the differentiation progress of skeletal muscle cells was induced by high temperature.

In addition, an in vitro wound scratch assay was performed to evaluate the effect of temperature on cell migration. The wound closure rates of cells cultured at 21 °C, 25 °C, and 28 °C were calculated at different times postwounding. The results showed that the wound closure rates increased remarkably with increasing temperature, although the difference between cells cultured at 25 °C and 28 °C was not significant at 24 h postwounding (Figure 1F,G). Overall, the above results indicated that higher temperatures significantly accelerated the proliferation, differentiation, and migration of skeletal muscle cells.

### 2.2. Statistics of RNA-Seq Data

A total of 18 sequencing libraries were constructed for the skeletal muscle cells at proliferation (PH1, PH2, PH3, PM1, PM2, PM3, PL1, PL2, PL3) and differentiation (DH1, DH2, DH3, DM1, DM2, DM3, DL1, DL2, DL3) stages, which were cultured at 28 °C (H), 25 °C (M) or 21 °C (L), respectively, producing a total of 41.61 million raw reads. After filtering, a total of 39.38 million clean reads were obtained, of which 88.68–94.62% for each library was mapped to the spotted sea bass genome (Table S1).

### 2.3. Comparative Transcriptome Analysis between Cells in the Proliferation and Differentiation Stages

**Figure 2:**
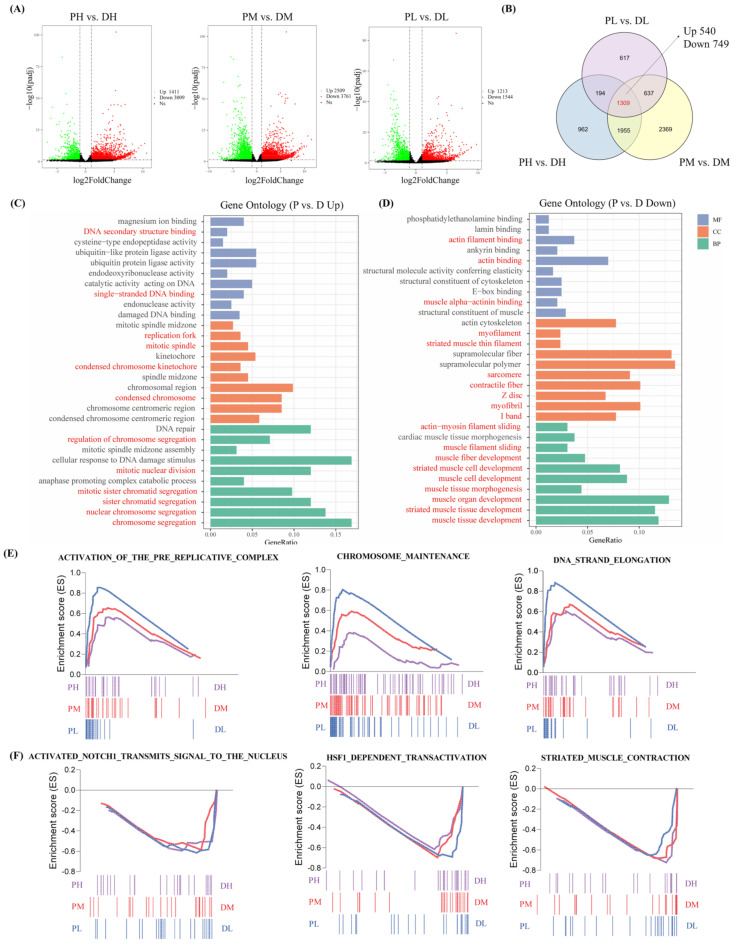
Comparative transcriptome analysis between cells in the proliferation and differentiation stages. (**A**) Venn diagram of DEGs between the proliferation and differentiation comparison groups (PH vs. DH, PM vs. DM, and PL vs. DL). (**B**) Volcano plots of DEGs between the proliferation and differentiation comparison groups (PH vs. DH, PM vs. DM, and PL vs. DL). (**C**) Significantly upregulated GO terms in the proliferation group, the GO terms related to DNA replication process were shown in red. (**D**) Significantly upregulated GO terms in the differentiation group, the GO terms related to myogenesis process were shown in red. The vertical axis represents the molecular function (MF), cellular component (CC), and biological processes (BP) terms, and the horizontal axis represents the gene proportion enriched in the corresponding terms. (**E**) GSEA plots showing the significantly enriched gene sets positively correlated with the proliferation group. (**F**) GSEA plots showing the significant enriched gene sets positively correlated with the differentiation group.

Principal component analysis (PCA) of the gene expression was conducted to evaluate the association between samples. Results showed that in the PCA plot, the replicated samples within the same group were located close to each other, and samples of different groups were positioned spatially distinct (Figure S1), indicating the reliability of our experiment. Gene expression levels were initially compared between cell samples at the two developmental stages (proliferation vs. differentiation), which were cultured at the same temperature. Therefore, three pairwise comparison groups were generated, including PL vs. DL, PM vs. DM, and PH vs. DH groups. As shown in Figure 2A, there were 1411 upregulated and 3009 downregulated differentially expressed genes (DEGs) in the PH groups compared with the DH group, 2509 upregulated and 3761 downregulated DEGs between the PM vs. DM groups, and 1213 upregulated and 1544 downregulated DEGs between the PL vs. DL groups. Specifically, a total of 1309 overlapping DEGs among the three comparison groups, including 540 upregulated and 749 downregulated genes in the proliferation groups compared with the differentiation groups, were identified (Figure 2B). GO enrichment analysis was conducted for those 1309 common DEGs to provide insights into their potential functions. As shown in Figure 2C, upregulated DEGs in the proliferation stage compared with the differentiation stage were mainly enriched in DNA replication-related GO terms, such as single-stranded DNA binding, DNA secondary structure binding in molecular function (MF), replication folk, mitotic spindle, condensed chromosome kinetochore, and condensed chromosome with respect to the cellular component (CC) and regulation of chromosome segregation, mitotic nuclear division, mitotic sister chromatid segregation, sister chromatid segregation, nuclear chromosome segregation, and chromosome segregation with respect to biological processes (BP). In contrast, downregulated DEGs in the proliferation stage compared with the differentiation stage were significantly enriched in myogenic differentiation-related GO terms, including actin binding, actin filament binding, muscle alpha-actin binding in MF, myofilament, striated muscle thin filament, sarcomere, contractile fiber, Z disc, myofibril, I band, and myofilament with respect to CC and actin-myosin filament sliding, muscle filament sliding, muscle fiber development, striated muscle cell development, muscle cell development, muscle tissue morphogenesis, muscle organ development, striated muscle tissue development, and muscle tissue development with respect to BP (Figure 2D).

The gene set enrichment analysis (GSEA) was performed to extract further biological insights from the whole gene expression datasets instead of limiting by the arbitrary threshold for the identified DEGs. Overall, DNA replication-related gene sets, such as DNA strand elongation, chromosome maintenance, and activation of the prereplicative complex, were significantly positively correlated with proliferation groups (gene sets that were upregulated in PH, PM, and PL) (Figure 2E). Meanwhile, gene sets including striated muscle contraction, HSF1-dependent transactivation and activated NOTCH1 transmits signal to the nucleus were positively correlated with the differentiation group (gene sets that were downregulated in PH, PM, and PL) (Figure 2F).

### 2.4. Identification of Significant Modules and Genes Associated with Temperature by WGCNA

**Figure 3:**
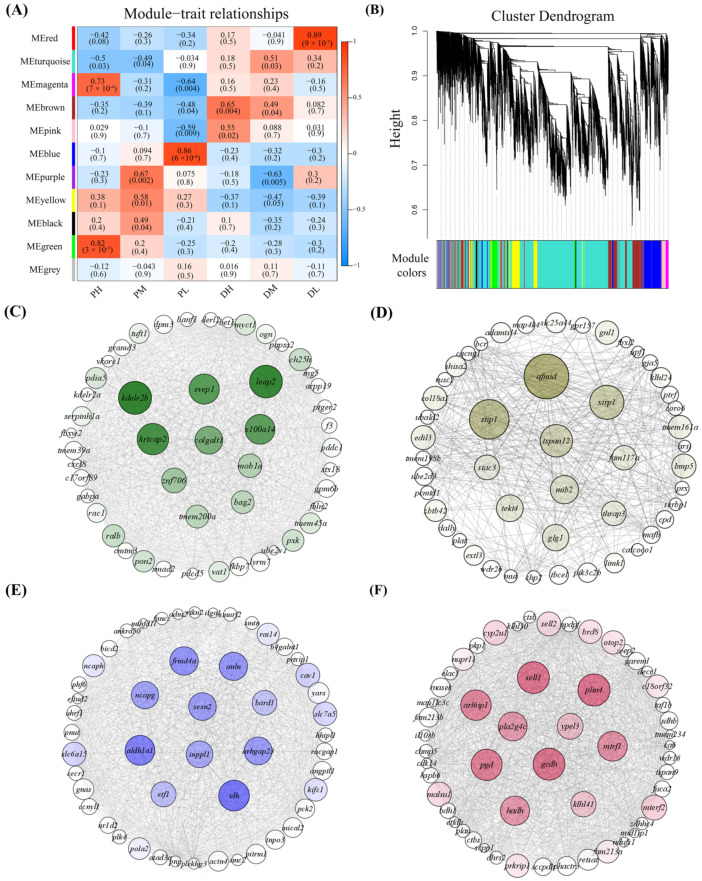
Identification of key modules and genes associated with temperature at proliferation and differentiation stages by WGCNA. (**A**) Gene dendrogram based on the TOM Matrix. Each colored row represents a color-labeled module that contains a group of highly related genes. (**B**) The heatmap of the relationship between modules and traits. The value above the box represents the correlation coefficient, and the value below the box represents the *p*-value. (**C**) PPI network of DEGs and the identified hub genes in the green module which were associated with the PH trait. (**D**) PPI network of DEGs and the identified hub genes in the brown module which were associated with the DH trait. (**E**) PPI network of DEGs and the identified hub genes in the blue module which were associated with the PL trait. (**F**) PPI network of DEGs and the identified hub genes in the red module which were associated with the DL trait. The full names of genes are listed in Table S2.

Weighted gene co-expression network analysis (WGCNA) was performed to identify modules and genes highly correlated with temperature treatment. After filtering out the low-expressed genes (FPKM < 1) in 18 transcriptome sequencing libraries, 12,191 genes were retained for WGCNA. As a result, a total of 11 coexpression modules were generated, which are labeled using different colors in Figure 3A and are described on the y-axis of Figure 3B. Module–trait correlation calculation results indicated that four modules (green, blue, brown, and red modules) were significantly associated with temperature at proliferation or differentiation stages (PH, PL, DH, and DL groups); therefore, we selected these four modules for further analysis. To investigate the relationships of coexpressed genes and to identify key regulatory genes (hub genes), PPI networks were constructed by Cytoscape using the top 50 genes ranked by connectivity within each selected module. In detail, the green module was significantly correlated with the PH group (correlation coefficient = 0.82, *p*-value < 0.05). In total, 447 genes were included in this module, and *kdelr2b*, *leap2*, *krtcap2*, *svep1*, *s100a14*, *colgalt1*, *znf706*, *mob1a*, *tmem200a*, and *bag2* were identified as the hub genes (Figure 3C). For the blue module, which correlated with the PL group (correlation coefficient = 0.86, *p*-value < 0.05), a total of 1376 genes were included, and the *frmd4a*, *anln*, *ncapg*, *sesn2*, *bard1*, *aldh1a1*, *inppl1*, *arhgap23*, *etf1*, and *tdh* genes were identified as hub genes (Figure 3D). For the brown module, which was closely related to the DH group (correlation coefficient = 0.65, *p*-value < 0.05), a total of 1168 genes were included in this module, and *afmid*, *stip1*, *xirp1*, *tspan12*, *mib2*, *tekt4*, *glg1*, *stac3*, *fam117a*, and *thrap3* were recognized as hub genes (Figure 3E). For the red module, which was closely associated with the DL group (correlation coefficient = 0.89, *p*-value < 0.05), 320 genes in total were included, and *plin4*, *sell1*, *gcdh*, *pgd*, *arl6ip1*, *mtrf1*, *hadh*, *pla2g4c*, *klhl4l*, and *ypel3* were identified as hub genes (Figure 3F).

### 2.5. Functional Enrichment Analysis for Significant Modules Affected by Temperature

**Figure 4:**
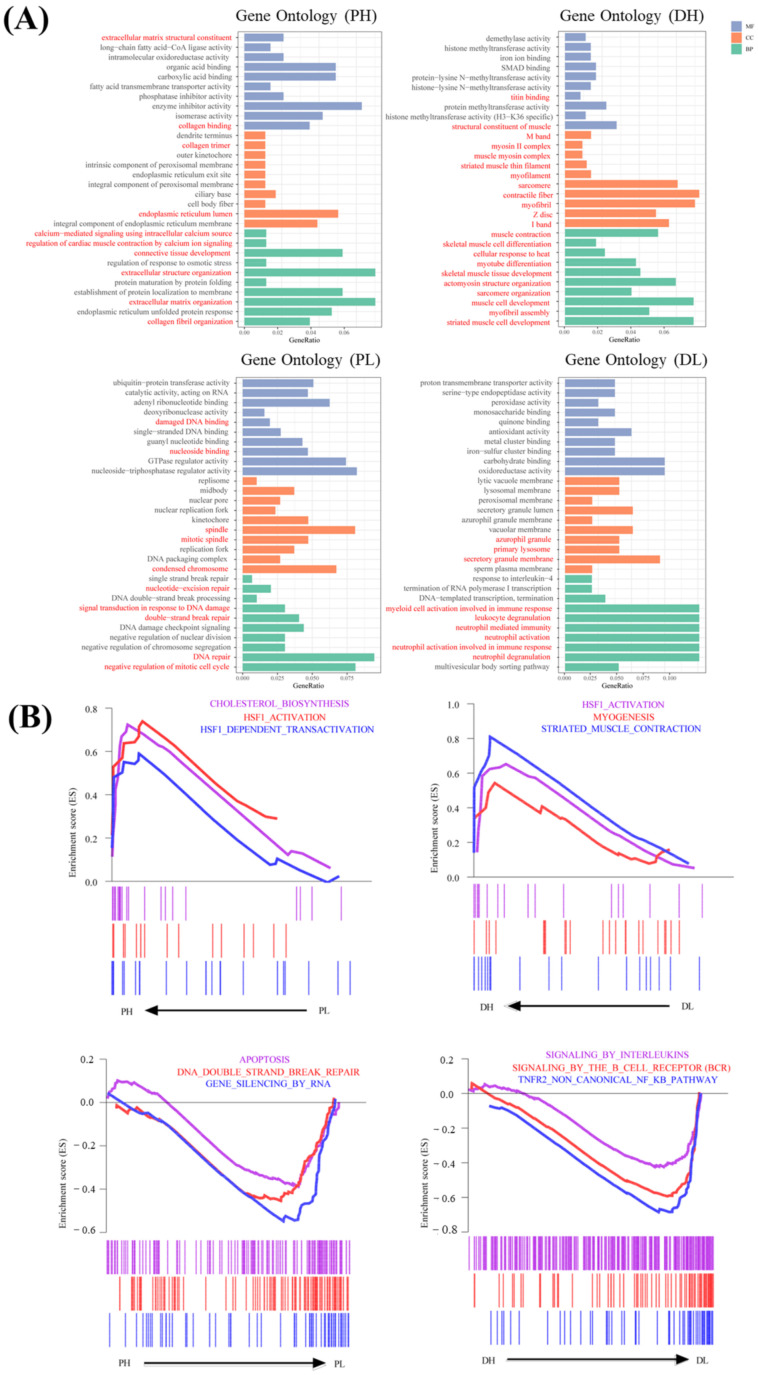
Functional enrichment analysis for genes affected by temperature. (**A**) GO enrichment analysis based on the genes from green, brown, blue and red modules associated with the PH, PL, DH, and DL traits, respectively. The vertical axis represents the molecular function (MF), cellular component (CC), and biological processes (BP) terms, and the horizontal axis represents the gene proportion enriched in the corresponding terms. (**B**) GSEA plots showing the significantly enriched gene sets positively correlated with the green (PH), brown (PL), blue (DH), and red (DL) modules, respectively.

To further investigate the biological functions related to the four significant modules, GO enrichment analysis was performed for the genes within each module. The results showed that genes in the green module (related to the PH group) were mainly enriched in extracellular matrix (ECM) organization-related GO terms, including extracellular matrix structural constituent and collagen binding in MF, collagen trimer in CC and extracellular structure organization, extracellular matrix organization, connective tissue development, and collagen fibril organization in BP. In addition, GO terms including endoplasmic reticulum lumen, integral component of endoplasmic reticulum membrane, calcium-mediated signaling using intracellular calcium source, and regulation of cardiac muscle contraction by calcium ion signaling were also significantly enriched in the PH group. Genes in the blue module (related to the PL group) were mainly enriched in DNA damage response- and repair-related GO terms, including damaged DNA binding and nucleoside binding in MF, mitotic spindle, spindle, and condensed chromosome with respect to CC, as well as double-strand break repair, nucleotide-excision repair, DNA repair, signal transduction in response to DNA damage and negative regulation of the mitotic cell cycle with respect to BP. In addition, genes in the brown module (related to the DH group) were mainly enriched in heat stress response, calcium homeostasis, myogenic differentiation, and sarcomere assembly-related GO terms, such as titin binding and structural constituent of muscle in MF, M band, myosin II complex, muscle myosin complex, striated muscle in thin filament, myofilament, sarcomere, contractile fiber, myofibril, Z disc, and I band with respect to CC, and muscle contraction, skeletal muscle cell differentiation, cellular response to heat, myotube differentiation, skeletal muscle tissue development, actomyosin structure organization, sarcomere organization, muscle cell development, myofibril assembly, and striated muscle cell development with respect to BP. Meanwhile, genes in the red module (related to the DL group) were mainly enriched in immune response-related GO terms, such as primary lysosome and azurophil granule with respect to CC, and myeloid cell activation involved in immune response, neutrophil-mediated immunity, neutrophil activation, neutrophil activation involved in immune response, leukocyte degranulation, and neutrophil degranulation with respect to BP (Figure 4A).

In addition, GSEA was performed to identify gene sets with a statistically significant difference between the PH and PL groups, as well as the DH and DL groups. The results showed that the most significantly enriched gene sets were positively correlated with the PH group, including HSF1 activation, HSF1-dependent transactivation, and cholesterol biosynthesis, while DNA double-strand break repair, apoptosis, and gene silencing by RNA were positively correlated with the PL group. For the DH group, the significantly enriched gene sets included myogenesis, striated muscle contraction, and HSF1 activation. The TNFR2 noncanonical NF-kB pathway, signaling by the B-cell receptor (BCR), and signaling by interleukins were positively correlated with the DL group (Figure 4B).

### 2.6. Putative Functional Roles of Genes in Skeletal Muscle Cells Affected by Temperature

**Figure 5:**
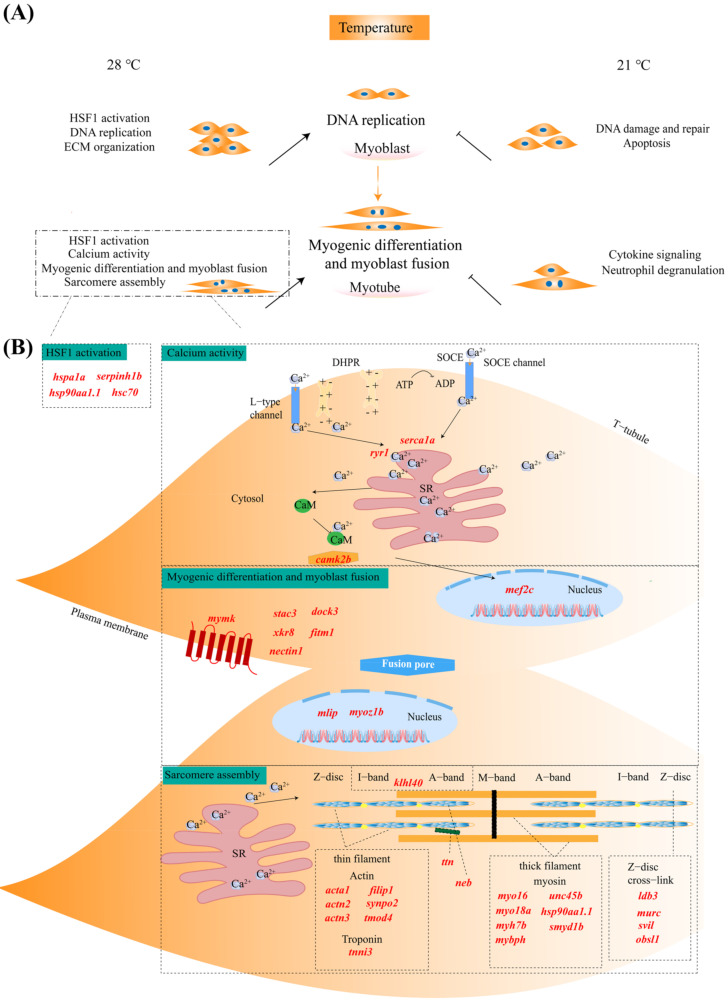
Schematic diagram of functional categories and associated key genes in skeletal myogenesis affected by temperature. (**A**) The putative schematic diagram of functional pathways involved in skeletal myogenesis regulated by temperature. (**B**) The key genes involved in skeletal myogenesis induced by high temperature (28 °C) at the differentiation stage. High temperature upregulated expressions of “HSF1 activation”, “Calcium activity”, “Myogenic differentiation and myoblast fusion”, and “Sarcomere assembly” related genes, respectively, which may account for the significantly accelerated developmental process of skeletal muscle cells. The DEGs identified in this study were labeled with red color. SR: sarcoplasmic reticulum. The full names of genes are listed in Table S2.

Based on the above functional enrichment analysis results, in combination with manual literature searches about the functions of DEGs, we summarized and drew a putative schematic diagram presenting the main functional categories affected by temperature at proliferation and differentiation stages (Figure 5A) and highlighted genes involved in skeletal myogenesis regulated by temperature (Figure 5B).

**Figure 6:**
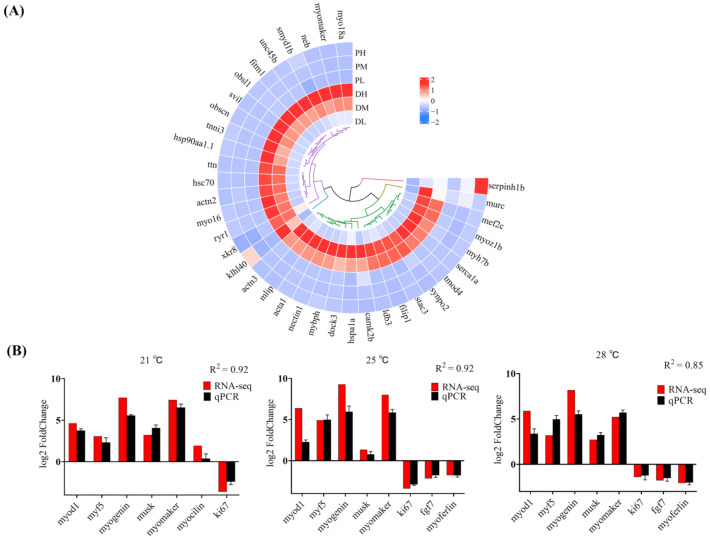
(**A**) Circos heatmap showing the expression values of genes involved in myogenesis affected by temperature. (**B**) Validation of RNA-seq data via qPCR. The x-axis displays 8 selected genes and Y-axis represents the fold change (Log2FC) value.

High temperature (28 °C) promoted “HSF1 activation”, “DNA replication” and “ECM organization”-related genes and pathways at the proliferation stage, which might be responsible for temperature sensation and promote the cell proliferation process (Figure 6A). In addition to “HSF1 activation”, it is worth noting that high temperature (28 °C) induced a large number of DEGs engaged in “Calcium activity”, “Myogenic differentiation and myoblast fusion”, and “Sarcomere assembly” at the differentiation stage, which may account for the significantly accelerated developmental process of skeletal muscle cells. In contrast, the most abundant functional classes affected by low temperature (21 °C) were “DNA damage and repair” and “Apoptosis” at the proliferation stage, as well as immune response-related pathways such as “Cytokine signaling” and “Neutrophil degranulation” at the differentiation stage (Figure 5A).

Furthermore, as we focus more on genes involved in myogenesis, the upregulated genes at high temperature were mainly classified into the following functional categories: HSF1 activation-mediated genes (heat shock proteins), including (1) *hspa1a*, *serpinh1b*, *hsp90aa1.1*, and *hsc70*; (2) calcium regulation-associated genes, including *ryr1*, *serca1a*, *camk2b*; (3) myogenic differentiation and myoblast fusion regulation-related genes, including *mef2c*, *mymk*, *stac3*, *mlip*, *nectin1*, *dock3*, *fitm1*, *myoz1b*, *xkr8*; and (4) sarcomere assembly-related genes, such as *acta1*, *actn2*, *actn3*, *filip1*, *synpo2*, *tmod4*, *tnni3*, *klhl40*, *ttn*, *neb*, *myo16*, *myo18a*, *myh7b*, *mybph*, *unc45b*, *hsp90aa1,1*, *smyd1b*, *ldb3*, *murc*, *svil*, and *obsl1* (Figure 5B). In addition, the expression values of the above subsets of genes are illustrated in Figure 6A, and their potential biological functions in promoting myogenesis progression under high temperature are discussed in detail in the Section 3.

### 2.7. Validation of RNA-Seq Data via qPCR

To validate the RNA-seq results, several DEGs (*myomaker*, *fgf7*, *musk*, *ki67*, *myocilin*, *myoferlin*, *myogenin*, *myod1*, and *myf5*) were selected for the qPCR analysis. Results showed that there was a positive correlation coefficient (R^2^ ≥ 0.85) between the RNA-seq and qPCR data, indicating that bioinformatic analysis results were reliable (Figure 6B).

## 3. Discussion

Changes in temperature have already attracted particular attention to the aquaculture industry because of their remarkable impact on muscle, which ultimately influences growth performance and meat quality [30,31]. Understanding the regulatory mechanism of temperature on skeletal muscle could facilitate the development of a rational management strategy to promote growth and optimize aquaculture production. However, the knowledge to date of the temperature-regulatory systems involved in controlling the skeletal muscle growth of fish is still limited. In this study, we utilized a cell culture method to investigate the effects of temperature on the skeletal muscle growth of spotted sea bass. At the cellular level, morphological observation provided direct evidence to characterize temperature function. When incubated at different temperatures (21 °C, 25 °C, and 28 °C), skeletal muscle cells exhibited a significant increase in cell number, wound healing rate, and cell fusion in response to rising temperatures, which indicated accelerated proliferation, differentiation, and migration rates at higher temperatures. This phenomenon was consistent with our previous in vivo study of juvenile spotted sea bass, which showed that the growth performance indicated by the parameters of both specific growth rate (SGR) and weight gain rate (WGR) was significantly higher when reared at 28 °C in comparison with 20 °C after 30 days [32]. The impacts of temperature on myogenesis have also been studied using a primary cultured muscle cell model in chickens, which reported the significant influence of thermal stress during myogenic proliferation and differentiation stages [33,34].

We then performed comparative transcriptome analysis to investigate the underlying molecular mechanism regulating the responses of skeletal muscle cells to different temperatures. As we summarized in Figure 5A, within the range of living temperature conditions, higher temperatures could improve the myogenic proliferation process mainly by activating the HSF1 pathway, altering ECM organization, and accelerating DNA replication. As the most well-known temperature-sensitive transcription factor, HSF1 and its downstream HSPs have been demonstrated to be essential regulators of cell proliferation in several cell types [35,36]. For example, it was proven that the signaling pathway activated by HSF1-Hsp27 facilitates Hek293 cell proliferation [37]. In pregnant rats, HSF1-HSP90 was localized in myometrial cells and suggested to play important roles in myometrial proliferation [38]. Moreover, cell proliferation mediated by Ras-induced ROS was recognized to be associated with cell type-specific alterations of the HSF1/SESN3/p21Cip1/WAF1 pathways [39]. HSF1 enhanced HeLa cell proliferation, migration, and invasion by enhancing MTDH-VEGF-C expression in vitro [40]. Although little is known about the roles of the HSF1 pathway in muscle cell proliferation, evidence has shown that the heat stress-associated increase in skeletal muscle mass in mice may be induced by HSF1 and/or the HSF1-mediated stress response that activates muscle satellite cells and the Akt/p70S6K signaling pathway [41].

The ECM is known to modulate cellular morphology and act as a conduit between extracellular stimuli and cells by regulating proliferation, migration, differentiation, and survival [42]. Skeletal muscle contains three layers of ECM, which include the endomysium, perimysium, and epimysium. These three layers of connective tissue structure provide both structural support and biochemical cues that direct muscle formation [43]. Interactions between skeletal muscle myoblasts and their ECM have been extensively documented. Liu et al. demonstrated by analyzing the protein interaction signals between cells that ECM could act on skeletal muscle progenitor cell proliferation [44]. Stern et al. confirmed that myoblasts cultured on ECM extracted from adult rat leg muscles have enhanced proliferation ability [45]. The results also demonstrated that ECM could have a direct effect on leiomyoma smooth muscle cells (LSMCs) and participate in their proliferation through the interplay between the collagen matrix and the PDGF-stimulated MAPK pathway [46].

During skeletal myogenesis, myoblasts irreversibly exit the cell cycle and differentiate and then fuse into multinucleated myotubes, which further mature into functional myofibers [4,47]. In our study, after 48 h of cultivation in differentiation medium, the skeletal muscle cells cultured at higher temperatures exhibited increased numbers of multinucleated myotubes (Figure 1D). Consistent with the phenotypic observation, the RNA-seq analysis results revealed that numerous DEGs engaged in myogenic differentiation and myoblast fusion leading to myotube formation showed upregulated expression levels at higher temperatures. The associated DEGs and their potential involvement in myogenesis are presented schematically in Figure 5B and discussed as follows: (1) HSF1 activation-mediated genes (heat shock proteins), (2) genes mediating calcium activity, (3) genes mediating myogenic differentiation and myoblast fusion, and (4) genes mediating sarcomere assembly.

In this study, *hspa1a* and *hsc70* belonging to the Hsp70 family, *hsp90aa1.1*, and *serpinh1b* (*hsp47*) were all upregulated at 28 °C compared with those at 21 °C. Evidence has well-confirmed that Hsp90α1 is required for organized myofibril assembly in skeletal muscles of zebrafish embryos through facilitating myosin folding and assembly into organized myofibril filaments [48]. In terms of muscular adaptation, Hsp70 has been demonstrated to play a pivotal role in preventing apoptosis, influencing energy metabolism, and facilitating cellular processes after heat treatment [49]. Although the function of hsp47 in skeletal myogenesis during adaptation to temperature changes has not been explored for ectothermic teleosts, as a collagen-specific molecular chaperone, the expression of Hsp47 in rat skeletal muscle may regulate collagen production with gravitational conditions [50]. Therefore, we speculated that higher temperature within the range of suitable living conditions may activate HSPs, which not only function as the heat sensor but also accelerate the skeletal myogenesis process via facilitating myofibril assembly and maintaining cellular homeostasis.

The process of myoblast fusion during skeletal myogenesis is calcium regulated, depending on both the continuous presence of cell surface calcium and an adequate intracellular Ca^2+^ [51]. Ca^2+^ stores are essential for eliciting a precise spatiotemporal pattern of Ca^2+^ signal in developing muscle cells [52]. In our study, *ryr1*, *serca1a,* and *camk2b* showed significantly higher expression levels at 28 °C than at 21 °C. RyR-mediated Ca^2+^ dynamics have been demonstrated as a universal requirement for skeletal myogenesis [53]. *Ryr1* is responsible for releasing Ca^2+^ from the SR to the myoplasm and *serca1a* functions to uptake Ca^2+^ from the myoplasm to the SR which maintains calcium homeostasis [53,54,55,56,57,58]. After the intracellular Ca^2+^ concentration reaches a certain threshold value, subsequently activating *camk2b* and forming the Ca^2+^/CaM complex, further inhibiting histone deacetylase II (HDACII) and increasing nuclear abundance of myogenin and myocyte enhancer factor 2 (MEF2) [59], which synergistically regulate myogenesis and induce the development of skeletal muscle [60,61].

Myogenic specification and differentiation are coordinated by a series of myogenic transcription factors. Two key transcription factor families including the myogenic basic helix-loop-helix (bHLH) proteins and myocyte enhancer factor 2 (MEF2) are essential for both specification and differentiation of muscle progenitors [62]. Previous studies have shown that MEF2 factors are essential for muscle differentiation, and MyoD and MEF2 family members function combinatorially to activate transcription and myogenesis [63]. Additionally, *mef2c* has been proved as a direct transcriptional target of the bHLH proteins during skeletal muscle development [63]. In our results, the expression of *mef2c* showed upregulation at 28 °C compared with those at 21 °C. Besides, muscle-specific genes such as the *myomaker*, *fitm1*, *stac3*, *mlip*, *myoz1b*, and *xkr8,* which are activated transcriptionally by myogenic transcription factors via binding to their conserved E-box (CANNTG) and then trigger the myoblast fusion process [64,65,66,67,68,69,70], all exhibited higher expression levels at 28 °C than those at 21 °C. The induced expressions of these key genes regulating myogenesis might be the key cause for the accelerated skeletal muscle myogenic differentiation and myoblast fusion process at higher temperature.

The sarcomere comprises precisely organized filament systems that include thin, thick, titin, and nebulin [71]. In our results, we identified a number of sarcomere assembly-associated genes such as *acta1*, *actn2*, *actn3*, *filip1*, *synpo2*, *tmod4*, *tnni3*, *myo16*, *myo18a*, *myh7b*, *mybph*, *unc45b*, *hsp90aa1,1*, *smyd1b*, *ldb3*, *murc*, *svil*, *obsl1 klhl40*, *ttn*, and *neb*, were all upregulated at 28 °C compared with those at 21 °C. For thin filament, *acta1*, *actn2*, and *actn3* [72] were considered as actin filament cross-linkers; *filip1* [73] and *synpo2* [74] were recognized as actin-binding proteins; *tnni3* [74] encoded a constituent protein of the troponin complex; *tmod4, klhl40* [75,76] located on the thin filament; *neb* was essential for maintaining proper thin-filament lengths. For thick filament, *myo16*, *myo18*, *myh7b*, *mybpc*, and *mybph* are the main components [77]. In addition, myosin chaperones including *unc45b, hsp90aa1.1*, and *smyd1b* play essential roles in the myofibril assembly [78,79]. For Z-disc cross-link, *murc* [80], *ldb3* [80], *obsl1*, and *svil* [81] are localized at the Z-line region of the sarcomere and function in maintaining the Z-disc integrity in skeletal myofibrils.

WGCNA was conducted to further identify the potential key regulators modulating the accelerated myogenesis process in response to higher temperatures. Our results revealed that *kdelr2b*, *leap2*, *krtcap2*, *svep1*, *s100a14*, *colgalt1*, *znf706*, *mob1a*, *tmem200a*, and *bag2* were identified as hub genes of the PH trait-associated module. Although their functions in myogenesis have not been explored, many of these genes were reported to contribute to the cell proliferation process through different signaling pathways, such as the Akt-mTOR/GSK3β, PI3K/AKT, and ERK1/2 signaling pathways [82,83,84,85,86,87,88,89]. In addition, *afmid*, *stip1*, *xirp1*, *tspan12*, *mib2*, *tekt4*, *glg1*, *stac3*, *fam117a*, and *thrap3* were identified as hub genes of the DH trait-associated module. Several genes, including *mib2* and *stac3* functions to myotube formation, have been well explored in vertebrate skeletal muscle cells [90,91,92], including founder myoblasts, and C2C12 myogenic cell line [86].

Overall, according to our transcriptomic analysis results, the significantly accelerated proliferation, differentiation, and migration rates of skeletal muscle cells in response to rising temperatures revealed by morphological observations (Figure 1) might result from the high temperature-induced expression levels of genes involved in “HSF1 activation”, “DNA replication” and “ECM organization” at the proliferation stage, as well as genes associated with “HSF1 activation”, “Calcium activity”, “Myogenic differentiation and myoblast fusion”, and “Sarcomere assembly” at the differentiation stage (Figure 5).

## 4. Materials and Methods

### 4.1. Ethics Statement

All experiments were conducted in accordance with the guidelines of the Animal Research and Ethics Committee of Ocean University of China (Permit Number: 2014201). In addition, our research did not involve endangered or protected species.

### 4.2. Tissue Isolation and Cell Culture

Healthy spotted sea bass weighing 28.46–31.35 g were anesthetized with 40 mg mL^−1^ 3-aminobenzoic acid ethyl ester methane-sulfonate (MS-222) and then wiped with 75% ethanol. The dorsal white skeletal muscle (1 cm^3^) was immediately taken and washed four times in phosphate-buffered saline (PBS) with 1% 100 U mL^−1^ penicillin and 100 µg mL^−1^ streptomycin (Solarbio, Beijing, China). Next, the skeletal muscle tissues were cleaned by removing adipose tissues, cut into small pieces (1 mm^3^), evenly distributed into a 175 cm^2^ standard cell culture chamber, supplemented with growth medium (GM) (L-15 medium (G-CLONE, Beijing, China) mixed with 20% fetal bovine serum (FBS, ABSIN, Shanghai, China), 10 ng mL^−1^ bFGF (Solarbio, Beijing, China), 1% 100 U mL^−1^ penicillin and 100 µg mL^−1^ streptomycin), and then cultured at 25 °C in a CO_2_-free incubator (Jinghong, Shanghai, China). Every 3–4 days, the whole medium was replaced with fresh medium until cell emigration and passaging. When the primary cells grew to approximately 70% abundance, 0.25% trypsin (Servicebio, Wuhan, China) was used for subculture, and the isolated cell suspension was precultured in the incubator for 2 h to remove the fibroblasts.

- Cell proliferation at different temperatures. The isolated cells were plated at a density of 15,000 cells per well in culture flasks and incubated in a 25 °C incubator with GM for 24 h. A subset of cells was divided into three parts of equal amounts and cultured at experimental temperatures of 21 °C, 25 °C, and 28 °C. The GM was changed every 24 h. After 72 h of proliferation, the cell medium was removed, and the cells were rinsed with cold PBS and collected for RNA extraction (Figure S2A).
- Cell differentiation at different temperatures. The other part of the isolated cells was divided into three equal parts and cultured with GM for 72 h of proliferation at 25 °C, reaching approximately 90% confluency. Then, the GM was replaced with differentiation medium (DM) (L-15, 2% horse serum (G-CLONE, Beijing, China), 1% 100 U mL^−1^ penicillin, and 100 µg mL^−1^ streptomycin) to induce differentiation, and the cells were cultured in incubators at 21 °C, 25 °C, and 28 °C. The DM was changed every 24 h. After 48 h of differentiation, the cell medium was removed, and the cells were rinsed with cold PBS and collected for RNA extraction (Figure S2B).

### 4.3. Cell Proliferation Assay

The effect of temperature on cell proliferation was measured by 5-ethynyl-2′-deoxyuridine (EdU) (Beyotime, Shanghai, China) and cell counting kit-8 (CCK-8) (Vazyme, Nanjing, China) assays with 3 replicates according to the manufacturer’s instructions.

In brief, for the EdU assay, the cells were divided into three equal parts and placed into three 6-well cell culture plates cultured in a 25 °C incubator overnight with GM. EdU (10 µM) was added to each well, and the cells were incubated for 6 h at 21 °C, 25 °C, and 28 °C, fixed with 4% PFA for 15 min, permeabilized in 0.25% Triton X-100 for 10 min, and reacted with Click-iT reaction buffer for 30 min. Subsequently, the images were visualized by an Echo Revolve microscope (ECHO, Chicago, IL, USA). The percentage was calculated as the ratio of the number of EdU-positive cells to the number of total nuclei stained with 4′,6-diamidino-2-phenylindole (DAPI, 2 mg/mL) in the image. Three microscopic vision fields of each well were randomly selected to calculate the ratio of positive cells.

For the CCK-8 assay, cells were divided into three equal parts, placed into three 96-well cell culture plates (5000 cells/well), and cultured in a 25 °C incubator overnight with GM. Subsequently, the cells were transferred and cultured at 21 °C, 25 °C, and 28 °C for 24 h, and then 10 µL CCK-8 reagent was added to the cell plate for 4 h. The capacity of cell proliferation was assessed by detecting the optical density (OD) of each well at 450 nm wavelength using a microplate reader (Bio-Rad, Hercules, CA, USA).

### 4.4. Cell Differentiation Assay

The effect of temperature on cell differentiation was measured by nuclear fusion index (NFI) assay. Cells were divided into three equal parts, placed into three 6-well cell culture plates, and cultured with GM for 72 h of proliferation at 25 °C until reaching approximately 90% confluency. Then, the GM was replaced with DM. Cells were induced to differentiate at 21 °C, 25 °C, and 28 °C for 48 h, and then the cell nuclei were stained with DAPI.

Briefly, cells were washed with cold PBS, fixed in 4% paraformaldehyde in PBS solution for 20 min, permeabilized in 0.25% Triton X-100 for 15 min, stained with 2 mg/mL DAPI for 2 min, and washed in PBS three times for 5 min. Images were taken under a fluorescence microscope (ECHO, Chicago, America). NFI was calculated manually as a ratio of the number of nuclei inside myotubes to the number of total nuclei after myotube formation at 21 °C, 25 °C, and 28 °C, as previously described [93]. Three microscopic vision fields of each well were randomly selected to calculate the myotube NFI, and the experiment was repeated three times.

### 4.5. In Vitro Wound Scratch Assay

The wound scratch assay was employed to evaluate the migration rates of cells cultured at different temperatures. First, cells were divided into three equal parts and placed into three 6-well cell culture plates, supplemented with GM, and cultured at a 25 °C incubator until they reached full confluency. A sterile 200 μL pipette tip was used to create a uniform scratch wound on the monolayer of cells, and then the cell debris was removed by washing with PBS 3 times. The scratch cells were cultured in serum-free medium at 21 °C, 25 °C, and 28 °C. Images were captured at 0, 12, and 24 h with an Echo Revolve microscope (ECHO, Chicago, America), and the scratch closure rate was measured using ImageJ software (v2.1.0, Bethesda, MD, USA).

### 4.6. RNA Extraction and Transcriptome Sequencing

The total RNA of cells at proliferating and differentiating stages which were cultured at three experimental temperatures (21°C, 25°C, and 28°C) (Figure S2) was extracted by TRIzol reagent (Vazyme, Nanjing, China) according to the manufacturer’s instructions. The RNA concentration and quality were evaluated by Nanodrop 2000 (Nanodrop Technologies, Wilmington, DE, USA), and the RNA integrity was confirmed by the Agilent 2100 Bioanalyzer (Agilent Technologies, Santa Clara, CA, USA). A total of 18 sequencing libraries were constructed using the NEBNext^®^ Ultra™ RNA Library Prep Kit for Illumina^®^ (NEB, Ipswich, MA, USA) following the manufacturer’s instructions and index codes were added to each sample to attribute sequences. Then all the libraries were sequenced on an Illumina Nova-Seq 6000 platform, generating 150 bp paired-end reads. The RNA-seq data have been deposited in the NCBI SRA database under the Accession number: PRJNA859992.

### 4.7. Identification of Differential Expressed Genes (DEGs)

Clean reads were obtained from the RNA-seq raw reads by removing adapters, reads with more than 5% unknown nucleotides (“N”), and reads with more than 20% low-quality bases (Q value < 10) using trimmomatic (v0.39) [94]. Filtered clean reads were aligned to the reference genome of spotted sea bass (NCBI BioProject Number: PRJNA408177) using Hisat2 (v2.1.0) [95] with default parameters. The number of clean reads mapped to protein-coding genes in each sample was counted using FeatureCounts (v1.5.0) [96]. Raw counts data were imported into the DEseq2 R package (v1.16.1) for normalization and differential analysis, and DEGs were identified with the thresholds of |log2(fold change)| ≥ 1 and *q*-value < 0.05.

### 4.8. Functional Enrichment Analysis

Functional enrichment analysis was performed by Gene Ontology (GO) using cluster Profiler R package (v3.18.1) with the threshold of *p*-value < 0.05. The gene set enrichment analysis (GSEA) analysis was performed based on the c2.cp.reactome pathways database using GSEA (v4.1.0). The fragments per kilobase of transcript per million fragments mapped (FPKM) values normalized by Stringtie (v2.1.7) [97] were used for the calculation of normalized enrichment score (NES) in GSEA. |NES| > 1, false discovery rate (FDR) < 0.25 and Nominal (NOM) *p*-value < 0.05 were regarded as the significant thresholds.

### 4.9. Weighted Gene Co-Expression Network Analysis (WGCNA) and Hub Genes Identification

After filtering the genes with FPKM values lower than 1, the weighted gene co-expression networks were constructed by the WGCNA package (v1.47) in R [98]. FPKM values of genes were imported to constructed co-expression modules. Expression correlation coefficients of these imported genes were calculated, and the soft threshold β = 14 was selected to build gene networks using a scale-free topology model. Genes with highly similar expression patterns were clustered into branches, and modules were identified by the tree cut algorithm. To identify significant modules related to proliferation or differentiation stages under different temperatures, module eigengenes (MEs) were used to calculate the module–trait relationship. The intramodular connectivity, the gene trait significance absolute value (GS), and the gene module membership (MM) within each significant module were calculated to screen hub genes. Here, the top 10 connectivity genes with GS > 0.2 and MM > 0.8 were considered hub genes. The screened hub genes and interaction network for each related module were subsequently visualized by Cytoscape (v3.8.2) [99].

### 4.10. Validation of Gene Expression by Real-Time PCR

Real-time PCR was performed for validating the bioinformatic analysis results of RNA-seq data. A part of the RNA samples obtained from the above cell culture experiments was reverse-transcripted to cDNA using the HiScript III 1st Strand cDNA Synthesis Kit (+gDNA wiper) (Vazyme, Nanjing, China). Real-time PCR was conducted by Applied Biosystems 7300 machines (Applied Biosystems, Foster, CA, USA). Eight genes including *myomaker*, *fgf7*, *musk*, *ki67*, *myocilin*, *myoferlin*, *myogenin*, *myod1*, and *myf5* were chosen for validation, and α-tubulin was regarded as the reference gene. The specific primers of selected genes are listed in Table S3. The reaction system of 10 µL included 5 μL of ChamQ™ SYBR Color qPCR Master Mix (Vazyme, Nanjing, China), 1 μL of cDNA templates, 0.2 μL of each primer, and 3.6 μL of ddH_2_O. The qPCR procedure was performed as follows: 95 °C for 30 s and 40 cycles of 95 °C for 10 s, 60 °C for 30 s, and 72 °C for 30 s. All qPCR experiments were performed by three biological sample replicates with three technical replicates. Then the relative gene expression values were calculated by the 2^−^^△△^^Ct^ method.

### 4.11. Statistical Analysis

The differences among different groups were analyzed by one-way analysis of variance (ANOVA) followed by Duncan’s multiple range test. The difference was considered statistically significant for *p*-value < 0.05. Data were shown as the mean ± standard deviation (SD). All statistical analysis was performed by IBM SPSS Statistics 21.0 software (IBM SPSS, Turkey). The results were shown by GraphPad Prism software (v8.4.2) (GraphPad Software, San Diego, CA, USA).

## 5. Conclusions

In our study, we characterized cultured muscle cells of *L. maculatus* for the first time and investigated the impacts of different temperatures (21 °C, 25 °C, and 28 °C) on myoblast proliferation and differentiation at both the morphologic and molecular levels. The results of CCK-8, EdU, wound scratch, and nuclear fusion index assays revealed that higher temperatures significantly accelerated the proliferation, differentiation, and migration processes of myogenic cells in vitro compared to lower temperatures. Gene expression profiles were revealed by RNA-seq analysis. According to GO, GSEA, and WGCNA analyses, higher temperature induced HSF1-activation, DNA-replication, and ECM-organization processes at the proliferation stage; conversely, lower temperature induced DNA-damage, DNA-repair, and apoptosis processes. At the differentiation phase, higher temperatures induced HSF1-activation, calcium activity regulation, myogenic differentiation and myoblast fusion, and sarcomere assembly processes; conversely, lower temperatures induced cytokine-signaling and neutrophil-degranulation processes. Overall, the cell morphological observation and RNA-seq data presented here provide a significant improvement to our current understanding of how temperature affects *L. maculatus* skeletal muscle development and growth.

## Data Availability

Publicly available datasets were analyzed in this study. This data can be found here: PRJNA859992.

## Supplementary Materials

The following supporting information can be downloaded at: https://www.mdpi.com/article/10.3390/ijms23179812/s1.

## References

[1] Vélez E.J., Lutfi E., Azizi S., Perelló M., Salmerón C., Riera-Codina M., Ibarz A., Fernández-Borràs J., Blasco J., Capilla E. (2017). Understanding fish muscle growth regulation to optimize aquaculture production. Aquaculture.

[2] Mommsen T.P. (2001). Paradigms of growth in fish. Comp. Biochem. Physiol. B Biochem. Mol. Biol..

[3] Biga P.R., Goetz F.W. (2006). Zebrafish and giant danio as models for muscle growth: Determinate vs. indeterminate growth as determined by morphometric analysis. Am. J. Physiol. Regul. Integr. Comp. Physiol..

[4] Chal J., Pourquié O. (2017). Making muscle: Skeletal myogenesis in vivo and in vitro. Development.

[5] Buckingham M., Rigby P.W.J. (2014). Gene Regulatory Networks and Transcriptional Mechanisms that Control Myogenesis. Dev. Cell.

[6] Bryson-Richardson R.J., Currie P.D. (2008). The genetics of vertebrate myogenesis. Nat. Rev. Genet..

[7] Zammit P.S. (2017). Function of the myogenic regulatory factors Myf5, MyoD, Myogenin and MRF4 in skeletal muscle, satellite cells and regenerative myogenesis. Semin. Cell Dev. Biol..

[8] Shan T., Xu Z., Wu W., Liu J., Wang Y. (2017). Roles of Notch1 Signaling in Regulating Satellite Cell Fates Choices and Postnatal Skeletal Myogenesis. J. Cell. Physiol..

[9] Johnston I.A., Bower N.I., Macqueen D.J. (2011). Growth and the regulation of myotomal muscle mass in teleost fish. J. Exp. Biol..

[10] Steinbacher P., Marschallinger J., Obermayer A., Neuhofer A., Sänger A.M., Stoiber W. (2011). Temperature-dependent modification of muscle precursor cell behaviour is an underlying reason for lasting effects on muscle cellularity and body growth of teleost fish. J. Exp. Biol..

[11] Garcia de la serrana D., Devlin R.H., Johnston I.A. (2015). RNAseq analysis of fast skeletal muscle in restriction-fed transgenic coho salmon (*Oncorhynchus kisutch*): An experimental model uncoupling the growth hormone and nutritional signals regulating growth. BMC Genom..

[12] Melo L.H., Martins Y.S., Melo R.M.C., Prado P.S., Luz R.K., Bazzoli N., Rizzo E. (2019). Low salinity negatively affects early larval development of *Nile tilapia*, *Oreochromis niloticus*: Insights from skeletal muscle and molecular biomarkers. Zygote.

[13] Rossi G.S., Turko A.J., Wright P.A. (2018). Oxygen drives skeletal muscle remodeling in an amphibious fish out of water. J. Exp. Biol..

[14] Garcia de la serrana D., Vieira V.L.A., Andree K.B., Darias M., Estévez A., Gisbert E., Johnston I.A. (2012). Development Temperature Has Persistent Effects on Muscle Growth Responses in Gilthead Sea Bream. PLoS ONE.

[15] Johnston I.A., Lee H.T., Macqueen D.J., Paranthaman K., Kawashima C., Anwar A., Kinghorn J.R., Dalmay T. (2009). Embryonic temperature affects muscle fibre recruitment in adult zebrafish: Genome-wide changes in gene and microRNA expression associated with the transition from hyperplastic to hypertrophic growth phenotypes. J. Exp. Biol..

[16] Anastasiadi D., Díaz N., Piferrer F. (2017). Small ocean temperature increases elicit stage-dependent changes in DNA methylation and gene expression in a fish, the European sea bass. Sci. Rep..

[17] Furuichi Y., Kawabata Y., Aoki M., Mita Y., Fujii N.L., Manabe Y. (2021). Excess Glucose Impedes the Proliferation of Skeletal Muscle Satellite Cells Under Adherent Culture Conditions. Front. Cell Dev. Biol..

[18] Kelc R., Trapecar M., Vogrin M., Cencic A. (2013). Skeletal muscle-derived cell cultures as potent models in regenerative medicine research. Muscle Nerve.

[19] Millay D.P., O’Rourke J.R., Sutherland L.B., Bezprozvannaya S., Shelton J.M., Bassel-Duby R., Olson E.N. (2013). Myomaker is a membrane activator of myoblast fusion and muscle formation. Nature.

[20] Bi P., Ramirez-Martinez A., Li H., Cannavino J., McAnally J.R., Shelton J.M., Sánchez-Ortiz E., Bassel-Duby R., Olson E.N. (2017). Control of muscle formation by the fusogenic micropeptide myomixer. Science.

[21] Ciecierska A., Motyl T., Sadkowski T. (2020). Transcriptomic profile of primary culture of skeletal muscle cells isolated from semitendinosus muscle of beef and dairy bulls. Int. J. Mol. Sci..

[22] Kong X., Wang X., Li M., Song W., Huang K., Zhang F., Zhang Q., Qi J., He Y. (2021). Establishment of myoblast cell line and identification of key genes regulating myoblast differentiation in a marine teleost, *Sebastes schlegelii*. Gene.

[23] Metzger K., Dannenberger D., Tuchscherer A., Ponsuksili S., Kalbe C. (2021). Effects of temperature on proliferation of myoblasts from donor piglets with different thermoregulatory maturities. BMC Mol. Cell Biol..

[24] Clark D.L., Coy C.S., Strasburg G.M., Reed K.M., Velleman S.G. (2016). Temperature effect on proliferation and differentiation of satellite cells from Turkeys with different growth rates. Poult. Sci..

[25] Kumar A., Singh N., Goswami M., Srivastava J.K., Mishra A.K., Lakra W.S. (2016). Establishment and characterization of a new muscle cell line of zebrafish (*Danio rerio*) as an in vitro model for gene expression studies. Anim. Biotechnol..

[26] Landemaine A., Ramirez-Martinez A., Monestier O., Sabin N., Rescan P.Y., Olson E.N., Gabillard J.C. (2019). Trout myomaker contains 14 minisatellites and two sequence extensions but retains fusogenic function. J. Biol. Chem..

[27] Lu K.L., Cai L.S., Wang L., Song K., Zhang C.X., Rahimnejad S. (2020). Effects of dietary protein/energy ratio and water temperature on growth performance, digestive enzymes activity and non-specific immune response of spotted seabass (*Lateolabrax maculatus*). Aquac. Nutr..

[28] Shin M.K., Park H.R., Yeo W.J., Han K.N. (2018). Effects of Thermal Stress on the mRNA Expression of SOD, HSP90, and HSP70 in the Spotted Sea Bass (*Lateolabrax maculatus*). Ocean Sci. J..

[29] Cheng Y., Li X., Wang L., Lu K., Song K., Ai Q., Mai K., Zhang C. (2021). Effects of dietary arginine levels on growth, immune function of physical barriers and serum parameters of spotted seabass (*Lateolabrax maculatus*) reared at different water temperatures. Aquaculture.

[30] Person-Le Ruyet J., Mahé K., Le Bayon N., Le Delliou H. (2004). Effects of temperature on growth and metabolism in a Mediterranean population of European sea bass, Dicentrarchus labrax. Aquaculture.

[31] Guderley H. (2004). Metabolic responses to low temperature in fish muscle. Biol. Rev. Camb. Philos. Soc..

[32] Hu Y. (2019). Studies on the Mechanism and Physiological Response to Temperature Tolerance of Juvenile Spotted Sea Bass (Lateolabrax maculatus).

[33] Harding R.L., Halevy O., Yahav S., Velleman S.G. (2016). The effect of temperature on proliferation and differentiation of chicken skeletal muscle satellite cells isolated from different muscle types. Physiol. Rep..

[34] Siddiqui S.H., Subramaniyan S.A., Kang D., Park J., Khan M., Shim K. (2021). Modulatory effect of heat stress on viability of primary cultured chicken satellite cells and expression of heat shock proteins ex vivo. Anim. Biotechnol..

[35] Schöffl F., Prandl R., Reindl A. (1998). Update on Signal Transduction Regulation of the Heat-Shock Response. Plant Physiol..

[36] Jiang S., Tu K., Fu Q., Schmitt D.C., Zhou L., Lu N., Zhao Y. (2015). Multifaceted roles of HSF1 in cancer. Tumor Biol..

[37] Baldelli S., Limongi D., Coni C., Ciccarone F., Ciotti M., Checconi P., Palamara A.T., Ciriolo M.R. (2021). BK Polyomavirus Activates HSF1 Stimulating Human Kidney Hek293 Cell Proliferation. Oxid. Med. Cell. Longev..

[38] Bhatti M., Dinn S., Miskiewicz E.I., MacPhee D.J. (2019). Expression of heat shock factor 1, heat shock protein 90 and associated signaling proteins in pregnant rat myometrium: Implications for myometrial proliferation. Reprod. Biol..

[39] Zamkova M., Khromova N., Kopnin B.P., Kopnin P. (2013). Ras-induced ROS upregulation affecting cell proliferation is connected with cell type-specific alterations of HSF1/SESN3/p21Cip1/WAF1 pathways. Cell Cycle.

[40] Shi X., Deng Z., Wang S., Zhao S., Xiao L., Zou J., Li T., Tan S., Tan S., Xiao X. (2021). Increased hsf1 promotes infiltration and metastasis in cervical cancer via enhancing mtdh-vegf-c expression. OncoTargets Ther..

[41] Ohno Y., Egawa T., Yokoyama S., Nakai A., Sugiura T., Ohira Y., Yoshioka T., Goto K. (2015). Deficiency of heat shock transcription factor 1 suppresses heat stress-associated increase in slow soleus muscle mass of mice. Acta Physiol..

[42] Cloutier G., Sallenbach-Morrissette A., Beaulieu J.F. (2019). Non-integrin laminin receptors in epithelia. Tissue Cell.

[43] Zhang W., Liu Y., Zhang H. (2021). Extracellular matrix: An important regulator of cell functions and skeletal muscle development. Cell Biosci..

[44] Liu Y.-X., Wu B.-B., Gong L., An C.-R., Lin J.-X., Li Q.-K., Jiang D.-M., Jin K.-X., Mechakra A., Bunpetch V. (2019). Dissecting cell diversity and connectivity in skeletal muscle for myogenesis. Cell Death Dis..

[45] Stern M.M., Myers R.L., Hammam N., Stern K.A., Eberli D., Kritchevsky S.B., Soker S., Van Dyke M. (2009). The influence of extracellular matrix derived from skeletal muscle tissue on the proliferation and differentiation of myogenic progenitor cells ex vivo. Biomaterials.

[46] Koohestani F., Braundmeier A.G., Mahdian A., Seo J., Bi J.J., Nowak R.A. (2013). Extracellular matrix collagen alters cell proliferation and cell cycle progression of human uterine leiomyoma smooth muscle cells. PLoS ONE.

[47] Dumont N.A., Bentzinger C.F., Sincennes M.C., Rudnicki M.A. (2015). Satellite cells and skeletal muscle regeneration. Compr. Physiol..

[48] Du S.J., Li H., Bian Y., Zhong Y. (2008). Heat-shock protein 90α1 is required for organized myofibril assembly in skeletal muscles of zebrafish embryos. Proc. Natl. Acad. Sci. USA.

[49] Gao C.-Q., Zhao Y.-L., Li H., Sui W.-G., Yan H.-C., Wang X.-Q. (2015). Heat stress inhibits proliferation, promotes growth, and induces apoptosis in cultured Lantang swine skeletal muscle satellite cells. J. Zhejiang Univ. Sci. B.

[50] Oguro A., Sakurai T., Fujita Y., Lee S., Kubota H., Nagata K., Atomi Y. (2006). The molecular chaperone HSP47 rapidly senses gravitational changes in myoblasts. Genes Cells.

[51] Gehlert S., Bloch W., Suhr F. (2015). Ca^2+^-dependent regulations and signaling in skeletal muscle: From electro-mechanical coupling to adaptation. Int. J. Mol. Sci..

[52] Friday B.B., Mitchell P.O., Kegley K.M., Pavlath G.K. (2003). Calcineurin initiates skeletal muscle differentiation by activating MEF2 and MyoD. Differentiation.

[53] Meissner G. (2017). The structural basis of ryanodine receptor ion channel function. J. Gen. Physiol..

[54] Rassier D.E. (2017). Sarcomere mechanics in striated muscles: From molecules to sarcomeres to cells. Am. J. Physiol. Cell Physiol..

[55] Lee K.J., Hyun C., Woo J.S., Park C.S., Kim D.H., Lee E.H. (2014). Stromal interaction molecule 1 (STIM1) regulates sarcoplasmic/endoplasmic reticulum Ca^2+^-ATPase 1a (SERCA1a) in skeletal muscle. Pflugers Arch. Eur. J. Physiol..

[56] Stringer R.N., Jurkovicova-Tarabova B., Huang S., Haji-Ghassemi O., Idoux R., Liashenko A., Souza I.A., Rzhepetskyy Y., Lacinova L., Van Petegem F. (2020). A rare CACNA1H variant associated with amyotrophic lateral sclerosis causes complete loss of Cav3.2 T-type channel activity. Mol. Brain.

[57] Perestenko P., Watanabe M., Beusnard-Bee T., Guna P., McIlhinney J. (2015). The second C2-domain of copine-2, copine-6 and copine-7 is responsible for their calcium-dependent membrane association. FEBS J..

[58] Konig S., Béguet A., Bader C.R., Bernheim L. (2006). The calcineurin pathway links hyperpolarization (Kir2.1)-induced Ca^2+^ signals to human myoblast differentiation and fusion. Development.

[59] Benavides Damm T., Egli M. (2014). Calcium’s role in mechanotransduction during muscle development. Cell. Physiol. Biochem..

[60] Ridgeway A.G., Wilton S., Skerjanc I.S. (2000). Myocyte enhancer factor 2C and myogenin up-regulate each other’s expression and induce the development of skeletal muscle in P19 cells. J. Biol. Chem..

[61] Jin W., Liu M., Peng J., Jiang S. (2017). Function analysis of Mef2c promoter in muscle differentiation. Biotechnol. Appl. Biochem..

[62] Dodou E., Xu S.M., Black B.L. (2003). Mef2C Is Activated Directly By Myogenic Basic Helix-Loop-Helix Proteins during Skeletal Muscle Development in Vivo. Mech. Dev..

[63] Cong X., Doering J., Mazala D.A.G., Chin E.R., Grange R.W., Jiang H. (2016). The SH3 and cysteine-rich domain 3 (Stac3) gene is important to growth, fiber composition, and calcium release from the sarcoplasmic reticulum in postnatal skeletal muscle. Skelet. Muscle.

[64] Polster A., Nelson B.R., Olson E.N., Beam K.G. (2016). Stac3 has a direct role in skeletal muscle-type excitation-contraction coupling that is disrupted by a myopathy-causing mutation. Proc. Natl. Acad. Sci. USA.

[65] Reinholt B.M., Ge X., Cong X., Gerrard D.E., Jiang H. (2013). Stac3 Is a Novel Regulator of Skeletal Muscle Development in Mice. PLoS ONE.

[66] Bower N.I., Garcia De La Serrana D., Cole N.J., Hollway G.E., Lee H.T., Assinder S., Johnston I.A. (2012). Stac3 is required for myotube formation and myogenic differentiation in vertebrate skeletal muscle. J. Biol. Chem..

[67] Reid A.L., Wang Y., Samani A., Hightower R.M., Lopez M.A., Gilbert S.R., Ianov L., Crossman D.K., Dell’Italia L.J., Millay D.P. (2020). DOCK3 is a dosage-sensitive regulator of skeletal muscle and Duchenne muscular dystrophy-associated pathologies. Hum. Mol. Genet..

[68] Ren R.M., Liu H., Zhao S.H., Cao J.H. (2016). Targeting of miR-432 to myozenin1 to regulate myoblast proliferation and differentiation. Genet. Mol. Res..

[69] Kim G.W., Nam G.H., Kim I.S., Park S.Y. (2017). Xk-related protein 8 regulates myoblast differentiation and survival. FEBS J..

[70] Henderson C.A., Gomez C.G., Novak S.M., Mi-Mi L., Gregorio C.C. (2017). Overview of the muscle cytoskeleton. Compr. Physiol..

[71] Ebashi S., Ebashi F. (1965). Alpha-actinin, a new structural protein from striated muscle. I. Preparation and action on actomyosin-ATP interaction. J. Biochem..

[72] Militello G., Hosen M.R., Ponomareva Y., Gellert P., Weirick T., John D., Hindi S.M., Mamchaoui K., Mouly V., Döring C. (2018). A novel long non-coding RNA Myolinc regulates myogenesis through TDP-43 and Filip1. J. Mol. Cell Biol..

[73] Kai F.B., Fawcett J.P., Duncan R. (2015). Synaptopodin-2 induces assembly of peripheral actin bundles and immature focal adhesions to promote lamellipodia formation and prostate cancer cell migration. Oncotarget.

[74] Sheng J.-J., Jin J.-P. (2015). TNNI1, TNNI2 and TNNI3: Evolution, regulation, and protein structure–function relationships. Gene.

[75] Gokhin D.S., Lewis R.A., McKeown C.R., Nowak R.B., Kim N.E., Littlefield R.S., Lieber R.L., Fowler V.M. (2010). Tropomodulin isoforms regulate thin filament pointed-end capping and skeletal muscle physiology. J. Cell Biol..

[76] Garg A., O’Rourke J., Long C., Doering J., Ravenscroft G., Bezprozvannaya S., Nelson B.R., Beetz N., Li L., Chen S. (2014). KLHL40 deficiency destabilizes thin filament proteins and promotes Nemaline myopathy. J. Clin. Investig..

[77] Mouton J., Loos B., Moolman-Smook J.C., Kinnear C.J. (2015). Ascribing novel functions to the sarcomeric protein, myosin binding protein H (MyBPH) in cardiac sarcomere contraction. Exp. Cell Res..

[78] Etard C., Armant O., Roostalu U., Gourain V., Ferg M., Strähle U. (2015). Loss of function of myosin chaperones triggers Hsf1-mediated transcriptional response in skeletal muscle cells. Genome Biol..

[79] Armant O., Gourain V., Etard C., Strähle U. (2016). Whole transcriptome data analysis of zebrafish mutants affecting muscle development. Data Br..

[80] Tagawa M., Ueyama T., Ogata T., Takehara N., Nakajima N., Isodono K., Asada S., Takahashi T., Matsubara H., Oh H. (2008). MURC, a muscle-restricted coiled-coil protein, is involved in the regulation of skeletal myogenesis. Am. J. Physiol. Cell Physiol..

[81] Hedberg-Oldfors C., Meyer R., Nolte K., Rahim Y.A., Lindberg C., Karason K., Thuestad I.J., Visuttijai K., Geijer M., Begemann M. (2020). Loss of supervillin causes myopathy with myofibrillar disorganization and autophagic vacuoles. Brain.

[82] Geister K.A., Lopez-Jimenez A.J., Houghtaling S., Ho T.H., Vanacore R., Beier D.R. (2019). Loss of function of Colgalt1 disrupts collagen post-translational modification and causes musculoskeletal defects. DMM Dis. Model. Mech..

[83] Liu H., Zhu J., Mao Z., Zhang G., Hu X., Chen F. (2018). Tuft1 promotes thyroid carcinoma cell invasion and proliferation and suppresses apoptosis through the Akt-mTOR/GSK3β signaling pathway. Am. J. Transl. Res..

[84] Lin H., Zeng W., Lei Y., Chen D., Nie Z. (2021). Tuftelin 1 (TUFT1) Promotes the Proliferation and Migration of Renal Cell Carcinoma via PI3K/AKT Signaling Pathway. Pathol. Oncol. Res..

[85] Sun L., Chen G., Sun A., Wang Z., Huang H., Gao Z., Liang W., Liu C., Li K. (2020). BAG2 Promotes Proliferation and Metastasis of Gastric Cancer via ERK1/2 Signaling and Partially Regulated by miR186. Front. Oncol..

[86] Wei H., Ma W., Lu X., Liu H., Lin K., Wang Y., Ye Z., Sun L., Huang Z., Pan T. (2021). KDELR2 promotes breast cancer proliferation via HDAC3-mediated cell cycle progression. Cancer Commun..

[87] Zhu H., Gao W., Li X., Yu L., Luo D., Liu Y., Yu X. (2021). S100A14 promotes progression and gemcitabine resistance in pancreatic cancer. Pancreatology.

[88] Wang F., Xue Q., Xu D., Jiang Y., Tang C., Liu X. (2020). Identifying the hub gene in gastric cancer by bioinformatics analysis and in vitro experiments. Cell Cycle.

[89] Wang C., Wang Z., Zhang L., Lin X. (2021). MiR-29c inhibits the metastasis of oral squamous cell carcinoma and promotes its cell cycle arrest by targeting SERPINH1. Ann. Transl. Med..

[90] Carrasco-Rando M., Ruiz-Gómez M. (2008). Mind bomb 2, a founder myoblast-specific protein, regulates myoblast fusion and muscle stability. Development.

[91] Beckley S.J., Hunter M.C., Kituyi S.N., Wingate I., Chakraborty A., Schwarz K., Makhubu M.P., Rousseau R.P., Ruck D.K., de la Mare J.A. (2020). STIP1/HOP regulates the actin cytoskeleton through interactions with actin and changes in actin-binding proteins cofilin and profilin. Int. J. Mol. Sci..

[92] Nguyen H.T., Voza F., Ezzeddine N., Frasch M. (2007). Drosophila mind bomb2 is required for maintaining muscle integrity and survival. J. Cell Biol..

[93] Noë S., Corvelyn M., Willems S., Costamagna D., Aerts J.-M., Van Campenhout A., Desloovere K. (2022). The Myotube Analyzer: How to assess myogenic features in muscle stem cells. Skelet. Muscle.

[94] Bolger A.M., Lohse M., Usadel B. (2014). Trimmomatic: A flexible trimmer for Illumina sequence data. Bioinformatics.

[95] Kim D., Paggi J.M., Park C., Bennett C., Salzberg S.L. (2019). Graph-based genome alignment and genotyping with HISAT2 and HISAT-genotype. Nat. Biotechnol..

[96] Liao Y., Smyth G.K., Shi W. (2014). feature Counts: An efficient general purpose program for assigning sequence reads to genomic features. Bioinformatics.

[97] Pertea M., Pertea G.M., Antonescu C.M., Chang T.-C., Mendell J.T., Salzberg S.L. (2015). StringTie enables improved reconstruction of a transcriptome from RNA-seq reads. Nat. Biotechnol..

[98] Langfelder P., Horvath S. (2008). WGCNA: An R package for weighted correlation network analysis. BMC Bioinform..

[99] Reimand J., Isserlin R., Voisin V., Kucera M., Tannus-Lopes C., Rostamianfar A., Wadi L., Meyer M., Wong J., Xu C. (2019). Pathway enrichment analysis and visualization of omics data using g: Profiler, GSEA, Cytoscape and EnrichmentMap. Nat. Protoc..

