# Supplementary material for: Morphological and Molecular Responses of Lateolabrax maculatus Skeletal Muscle Cells to Different Temperatures
Source: Int J Mol Sci. 2022 Aug 29;23(17):9812. doi: 10.3390/ijms23179812 (PMC9456278; doi:10.3390/ijms23179812)
Supplement: Supplementary file 1 [file ijms-23-09812-s001.zip › ijms-1856396-Supplementary.pdf]

Figure S1. PCA plot from the transcriptome of spotted sea bass skeletal muscle cells at proliferation and differentiation stages, which were cultured at 28°C, 25°C or 21°C. Ellipses and shapes show clustering of the samples.

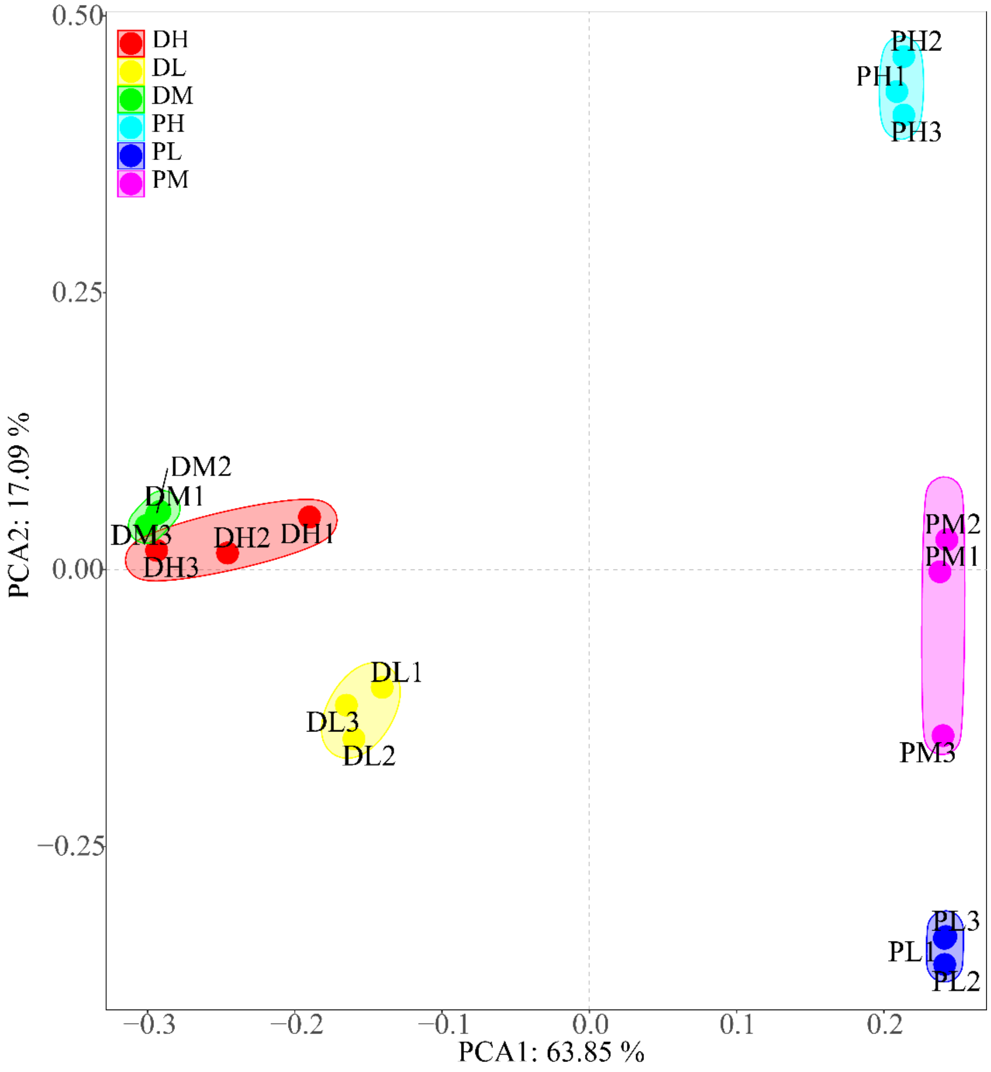

Figure S2. A schematic diagram of the experimental design for RNA-Seq sampling. (A) at the proliferation stage; **(B)** at the myogenic differentiation stages.

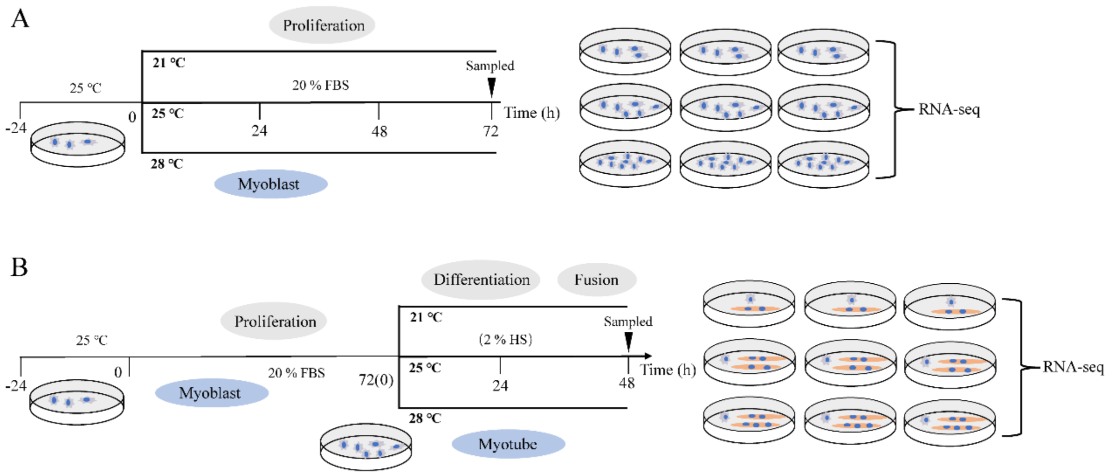

Table S1. The information of samples used for RNA-Seq

Sample	Raw Reads	Clean Reads	Mapping Clean Reads	Total mapping Ratio	Q20(%)	Q30(%)	GC Content(%)	Reads length(bp)
D-H-1	24130536	22211363	20352271.92	91.63%	96.55	91.73	40.66	150
D-H-2	23538538	23226909	21956397.08	94.53%	97.82	93.77	50.13	150
D-H-3	25104191	24189990	22349131.76	92.39%	97.48	93.34	49.51	150
D-M-1	27927734	26920163	24677713.42	91.67%	97.5	93.37	49.15	150
D-M-2	22631388	21826736	20108971.88	92.13%	97.53	93.41	49.42	150
D-M-3	20971291	20321039	18670970.63	91.88%	97.1	92.5	49.41	150
D-L-1	21039475	20373683	17725104.21	87.00%	97.42	93.16	48.26	150
D-L-2	23624801	22874066	21263731.75	92.96%	97.47	93.27	49.18	150
D-L-3	24414987	23756451	22072118.62	92.91%	97.6	93.5	49.26	150
P-H-1	21553131	19786881	18154463.32	91.75%	96.69	92.1	41.14	150
P-H-2	24081422	22045284	19549757.85	88.68%	95.63	90.58	37.53	150
P-H-3	23494948	21642011	19774305.45	91.37%	96.5	91.79	40.69	150
P-M-1	22113158	20523501	19214101.64	93.62%	97.21	92.86	41.36	150

P-M-2	22187142	20384007	19287347.42	94.62%	97.58	93.38	42.73	150
P-M-3	23454904	21235656	19891438.98	93.67%	97.14	92.53	38.88	150
P-L-1	20868948	19280224	18202459.48	94.41%	97.56	93.38	41.55	150
P-L-2	22521915	21546265	20193159.56	93.72%	97.88	94.07	48.28	150
P-L-3	22410401	21697101	20445178.27	94.23%	97.78	93.88	49.14	150

Table S2. Primers used for qPCR

Gene name	Primers sequences (5′to3′)
myod1-F	ACACCAGCGACATGCACTTCTTC
myod1-R	GAGGAGGAGGAGGAGGAGGAAGG
myf5-F	TCAACCACGCTTTAGAGGC
myf5-R	CGCCCAAACCGTCATTCT
myogenin-F	TCCATCCAGCCTGTCACCTCAC
myogenin-R	ACCTTCTTCAGACGCCTCTTCTCC
myomaker-F	CTGCGTTTCTACTTTGAGG
myomaker-R	GCATAGCGGTTCTTCTTT
myomixer-F	CTTGCTGCGGTCCCTGGTTATC
myomixer-R	AGAGGCTTCTCCTGAGGAACTGTG
ki67-F	CAGTGAGGCAGTCCAACGCTTC
ki67-R	GGGAGTTGTTACAGTGGTCGTCTTC
myoc-F	AGAGCCTGGAGGTGAAGAAGAGC
myoc-R	GGTGGTGTTGGTCGCAGTGTAG
myof-F	AGCGAGGAGCAGGACGACATAG
myof-R	ACGGTAGACCTTGAGGCTGAGTG
musk-F	ACTGCTCTGCCACTCCTCCTTC
musk-R	TGGACTGACCTGCTGCTCTCTAG
fgf7-F	TGAACTGCTCCAAACACG
fgf7-R	TATGCCGCCCTCAGACAC
*α-tubuli*n-F	AGGTCTCCACAGCAGTAGTAGAGC
α-tubulin-R	GTCCACCATGAAGGCACAGTCG

Note: *α-tubulin* was regarded as the reference gene.

Table S3. The list of full gene names

Gene name	Gene full name
kdelr2b	KDEL Endoplasmic Reticulum Protein Retention Receptor 2
leap2	Liver Enriched Antimicrobial Peptide 2

krtcap2	Keratinocyte Associated Protein 2
svep1	sushi, von Willebrand factor type A, EGF, and pentraxin domain-containing protein 1
s100a14	S100 Calcium Binding Protein A14
colgalt1	Collagen Beta(1-O)Galactosyltransferase 1
znf706	Zinc Finger Protein 706
mob1a	MOB Kinase Activator 1A
tmem200a	Transmembrane Protein 200A
bag2	BAG Cochaperone 2
frmd4a	FERM Domain Containing 4A
anln	Anillin
ncapg	Non-SMC Condensin I Complex Subunit G
sesn2	Sestrin 2
bard1	BRCA1 Associated RING Domain 1
aldh1a1	Aldehyde Dehydrogenase 1 Family Member A1
inppl1	Inositol Polyphosphate Phosphatase Like 1
arhgap23	Rho GTPase Activating Protein 23
etf1	Eukaryotic Translation Termination Factor 1
tdh	L-Threonine Dehydrogenase (Pseudogene)
afmid	Arylformamidase
stip1	Stress Induced Phosphoprotein 1
xirp1	Xin Actin Binding Repeat Containing 1
tspan12	Tetraspanin 12
mib2	MIB E3 Ubiquitin Protein Ligase 2
tekt4	Tektin 4
glg1	Golgi Glycoprotein 1
stac3	SH3 And Cysteine Rich Domain 3
fam117a	Family With Sequence Similarity 117 Member A
thrap3	Thyroid Hormone Receptor Associated Protein 3
plin4	Perilipin 4
sell1	Selectin L
gcdh	Glutaryl-CoA Dehydrogenase
pgd	Phosphogluconate Dehydrogenase
arl6ip1	ADP Ribosylation Factor Like GTPase 6 Interacting Protein 1
mtrf1	Mitochondrial Translation Release Factor 1
hadh	Hydroxyacyl-CoA Dehydrogenase
pla2g4c	Phospholipase A2 Group IVC
klhl4l	Kelch Like Family Member 4l
ypel3	Yippee Like 3
hspa1a	Heat Shock Protein Family A (Hsp70) Member 1A
hsc70	heat shock cognate protein 70

serpinh1b	Serpin Family H Member 1b
ryr1	Ryanodine Receptor 1
camk2b	Calcium/Calmodulin Dependent Protein Kinase II Beta
serca1a	sarco-endoplasmic reticulum Ca(2+)-ATPase
mef2c	Myocyte Enhancer Factor 2C
dock3	Dedicator Of Cytokinesis 3
stac3	SH3 And Cysteine Rich Domain 3
mymk	Myomaker
nectin1	Nectin Cell Adhesion Molecule 1
xkr8	XK Related 8
fitm1	Fat Storage Inducing Transmembrane Protein 1
mlip	Muscular LMNA Interacting Protein
myoz1b	Myozenin 1
klhl40	Kelch Like Family Member 40
ttn	titin
neb	nebulin
acta1	Actin Alpha 1
actn2	Actinin Alpha 2
actn3	Actinin Alpha 3
filip1	Filamin A Interacting Protein 1
synpo2	Synaptopodin 2
tmod4	Tropomodulin 4
tnni3	Troponin I3
myo16	Myosin XVI
myo18a	Myosin XVIIIA
myh7b	Myosin Heavy Chain 7B
mybph	Myosin Binding Protein H
unc45b	Unc-45 Myosin Chaperone B
hsp90aa1.1	Heat Shock Protein 90 Alpha Family Class A Member 1
smyd1b	SET And MYND Domain Containing 1
obscn	Obscurin
ldb3	LIM Domain Binding 3
murc	Muscle-Restricted Coiled-Coil
svil	Supervillin
obsl1	Obscurin Like Cytoskeletal Adaptor 1
